# Early milk-feeding regimes in calves exert long-term effects on the development of ovarian granulosa cells

**DOI:** 10.1186/s12864-023-09589-7

**Published:** 2023-08-25

**Authors:** Volker Röttgen, Lisa-Maria Tümmler, Dirk Koczan, Alexander Rebl, Björn Kuhla, Jens Vanselow, Anja Baufeld

**Affiliations:** 1 Research Institute for Farm Animal Biology (FBN), Wilhelm-Stahl-Allee 2, 18196 Dummerstorf, Germany; 2 Institute for Immunology, University of Rostock, 18055 Rostock, Germany

**Keywords:** Gene expression, Follicle count, Innate immune system, Interferon, Plane of nutrition

## Abstract

**Background:**

Nutrition has not only an impact on the general wellbeing of an animal but can also affect reproductive processes. In cattle, feeding regimes can influence the age of puberty onset and alter gonadal development. We analyzed effects of different milk replacer (MR) feeding regimes during rearing on ovarian physiology with specific emphasis on the numbers as well as gene expression characteristics of granulosa cells (GCs) at the age of puberty onset. Two groups of calves received either 10% or 20% of bodyweight MR per day during their first 8 weeks. After weaning, both groups were fed the same mixed ration ad libitum until slaughter at 8 months.

**Results:**

Animals of the 20% feeding group had a significantly higher body weight, but the proportion of animals having a *corpus luteum* at the time of slaughter was not different between groups, suggesting a similar onset of puberty. Calves of the 10% group showed a constant GC count regardless of the number of follicles (r = 0.23) whereas in the 20% group increasing numbers of GCs were detected with a higher follicle count (r = 0.71). As a first effort to find a possible molecular explanation for this unexpected limitation of GC numbers in the 10% group, we comparatively analyzed GC transcriptomes in both diet groups. The mRNA microarray analysis revealed a total of 557 differentially expressed genes comparing both groups (fold change > |1.5| and p < 0.05). *OAS1X*, *MX2* and *OAS1Z* were among the top downregulated genes in the 20% vs. the 10% group, whereas top upregulated genes comprised *BOLA* and *XCL1*. All of these genes are known to be regulated by interferon. Subsequent signaling pathway analysis revealed the involvement of several immune response mechanisms in accordance with a number of interferons as upstream regulators.

**Conclusions:**

The results indicate that the plane of MR feeding in early life has an impact on the number and physiology of GCs later in life. This might influence the overall reproductive life initiated by the onset of puberty in cattle. In addition, the observed alterations in GCs of calves fed less MR might be a consequence of interferon regulated immunological pathways.

**Supplementary Information:**

The online version contains supplementary material available at 10.1186/s12864-023-09589-7.

## Background

The onset of puberty in calves is an essential and economically important topic for the cattle industry as well as an indicator of reproductive fitness [1]. In general, a shorter generation interval and the introduction of new highly valuable breeding sires and heifers to breeding programs are main targets of breeding organizations. However, the onset of puberty is not only highly variable between different cattle breeds, it is also variable within a breed [2, 3]. Puberty is considered to depend on the one hand on breed genetics but on the other hand also on environmental factors [2, 4, 5]. Additionally, the process of sexual maturation depends on a complex network of biochemical processes in reproductive tissues along the hypothalamus-pituitary-gonadal axis [6]. Thus, the nutritional level appears to directly influence the LH pulse generating system in the hypothalamus with a high-energy diet increasing LH pulse frequency to attain puberty earlier [7, 8]. As nutrition affects the age of puberty onset, this leads to different feeding strategies in order to raise healthy animals with an early onset of puberty [8–13].

During conception the influence of different planes of nutrition have been studied in sheep resulting in phenotype alterations in male individuals [14]. The effect of different high protein and energy diets during the first trimester of pregnancy on the gonadal development of male offspring seems to have adverse effects [15]. In contrast, protein supplementation during late gestation in beef cows resulted in higher post-weaning body weight and higher pregnancy rates of female offspring [16].

In the early post-natal phase a higher plane of nutrition to female calves resulted not only in higher milk yields in the first lactation of dairy cows but also contributed to enhanced daily bodyweight gain as highlighted in a meta-analysis by Soberon and Van Amburgh and others [17, 18]. However, reliable findings about nutritional effects on female reproductive tissues, especially dairy cattle, are scarce. In young female beef calves, a high plane of nutrition resulted in enhanced development of reproductive tissues evidenced by increased weight of the reproductive tract at 3 month of age. In addition, calves fed a higher plane of nutrition were shown to have higher numbers of ovarian surface follicles and recovered oocytes [19]. In the same animal model, differentially expressed genes were detected in the hypothalamus, having consequences on the hypothalamic-pituitary-gonadal axis as shown by pathway analysis in this study thus implicating to affect reproductive development networks [20]. In addition, certain cell populations like visceral adipose tissue show alterations towards earlier reproductive functions [21].

In bull calves, increased food supply between weaning and the pubertal phase resulted in an earlier onset of puberty and larger testicles [9, 22, 23]. Whereas an increased energy supply during puberty positively affected testicular weight and size, it affected sperm production and quality negatively [24–26].

In females, theca and granulosa cells (GCs) play an important role for follicular development, steroid hormone production and oocyte maturation. The differentiation process of theca cells and GCs is orchestrated by the hypothalamus-derived gonadotropins FSH (follicle-stimulating hormone) and LH (luteinizing hormone). GCs and theca cells protect the oocyte from damaging systemic influences such as viral infections, increased concentrations of free fatty acids and heat stress, and are essential for the nutrient as well as oxygen supply of the oocyte [11, 12, 27]. Also diets differing in energy content influence the development of follicles in prepubertal beef heifers [28].

There is a growing body of evidence supporting the relevance of early life nutrition, and thus the crosstalk between metabolism and hypothalamic responses are important for molecular events associated with sexual maturation [20, 29]. However, these studies focus on alterations within the hypothalamus and to our knowledge, no reports exist about the nutritional effects during early life on the molecular level of the female reproductive tissues in dairy cattle. Hence, the aim of this study was to investigate effects of two different planes of early life nutrition on the ovaries of female Holstein calves considering the number of small- to medium sized follicles (< 6 mm) and granulosa cell numbers. In addition, we also analysed the granulosa cell transcriptomes and affected pathways in both diet groups.

## Results

### Effect of milk replacer supply on growth and ovarian differentiation

**Fig. 1:**
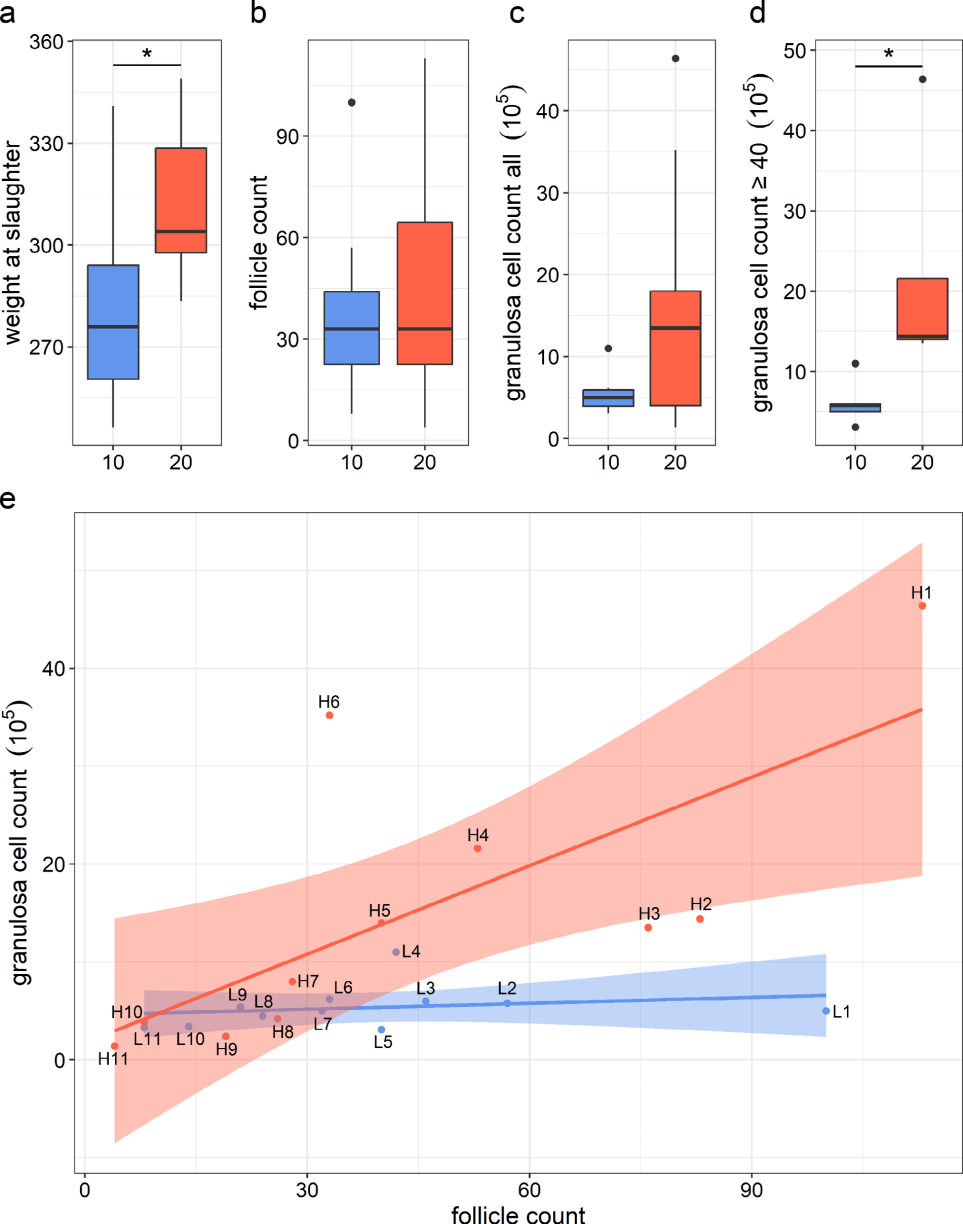
Different animal characteristics of the 10% (blue) and 20% (red) feeding group at time of slaughter. (**a**) The bodyweight (BW) at slaughter differed significantly between both feeding groups (indicated by asterisk, n = 11, t-test, p < 0.05). (**b**) Follicle cell count as well as (**c**) granulosa cell count of all animals were not significantly different (n = 11, t-test, p < 0.05). (**d**) The granulosa cell count of animals with 40 or more follicles differs significantly (n = 5, Mann-Whitney Rank test, p < 0.05). (**e**) The amount of GCs increased with increasing follicle numbers in the 20% feeding group (animals: H1-H11; r = 0.71, p < 0.05) but remained constant in the 10% feeding group (animals L1-L11; r = 0.23, p = 0.49)

As previously reported by Tümmler et al. [18] the bodyweight (BW) at slaughter ranged from 246 to 349 kg. The mean BW (Fig. 1a) of the 10% feeding group (279.8 kg ± 8.4) was significantly lower than of the 20% feeding group (312.5 ± 6.6) even though the birth weight was not significantly different (40 kg for the 10% group and 40.54 kg for the 20% group). Five out of 11 animals of the 10% group, had a *corpus luteum* (CL), whereas in the 20% group in eight out of 11 animals a CL was detected. According to the Fisher’s exact test this was not a statistically significant difference (p = 0.39), suggesting a similar onset of puberty. The amount of small to medium sized follicles (< 6 mm) was comparable in both groups (Fig. 1b). The GC count was lower in the 10% (5.3 × 10^5^) compared to the 20% group (15 × 10^5^) but due to increasing GC numbers with increasing follicle count only in the 20% group, this difference was not significant (Fig. 1c). In the 20% feeding group the total numbers of isolated living GCs positively correlated with the numbers of follicles (r = 0.71, p < 0.05; Fig. 1e). The 10% feeding group, however, did not show such a correlation (r = 0.23, p = 0.49). Instead, the numbers of isolated GCs did not increase with the numbers of follicles. To discover a possible cause for this unanticipated restriction of GC number in the 10% feeding group, we comparatively analyzed the transcriptomes of GCs in both diet groups. Especially the animals with a high follicle count (≥ 40) were clearly distinguishable between both feeding groups (Fig. 1d). The ratio of GC vs. follicle counts was significantly higher (p = 0.03) in the 20% feeding group (30,386 ± 5,348) than in the 10% feeding group (12,432 ± 3,687). Thus, only animals with a follicle count greater than or equal to 40 were selected for further analysis. Additionally, in this subset animals of both groups showed no significant difference (p = 0.52) in the presence of *corpora lutea*.

### Expression profiles of GCs after different early-life nutrition

**Fig. 2:**
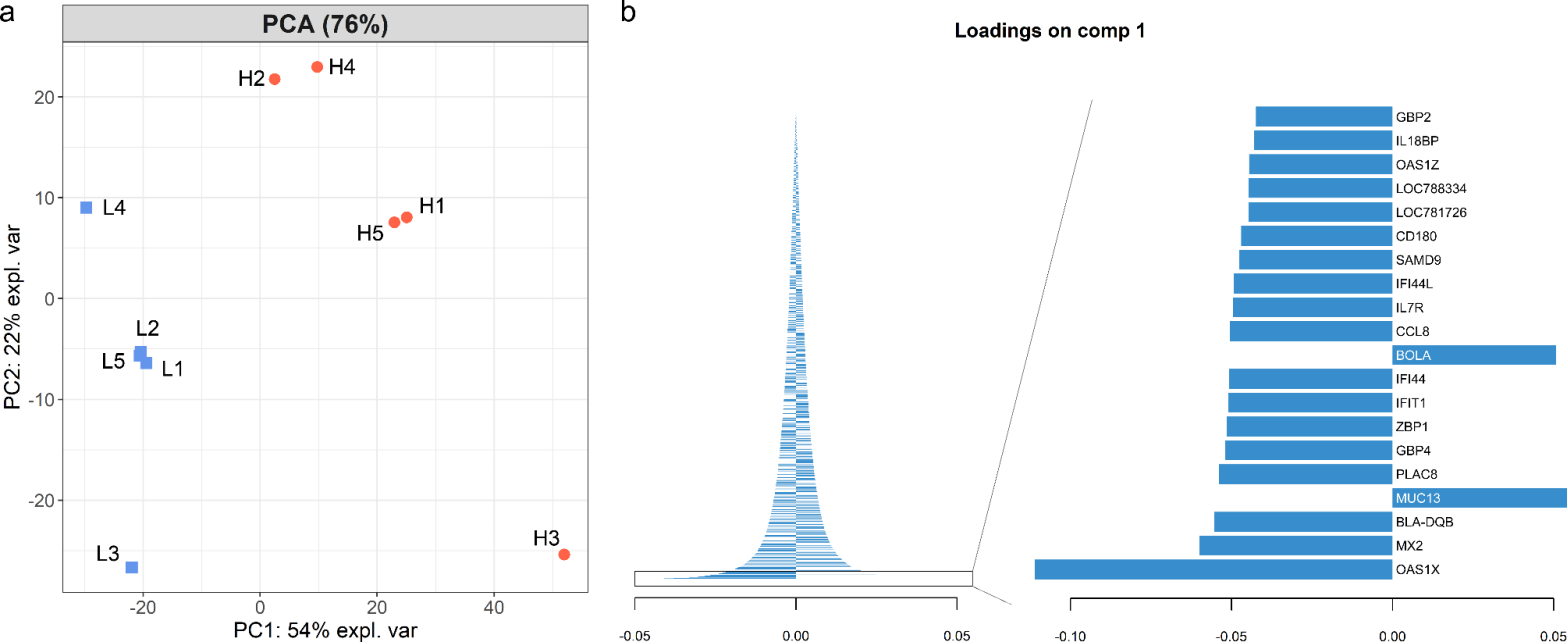
Principal component analysis (PCA) and loadings. In the PCA (**a**) each dot represents one animal either from the 10% (blue; L1-L5) or the 20% (red; H1-H5) feeding group. (**b**) The variable coefficients used to define the principal component 1 are shown while the length of each bar represents their importance. The left side of (**b**) displays the contribution of all variables. A more detailed information is obtained when only twenty variables are displayed (right)

The mRNA microarray dataset was subjected to a principal component analysis (PCA) to reduce the multidimensionality of the dataset (Fig. 2a). The maximum observed variance (PC1) could be obtained with 54.1%, whereas 21.9% of variance are displayed in the second component (PC2). Altogether, 76% of the variance of the data can be projected into the first two principal components (PCs), revealing that the greatest part of the information is retained. In the PCA ten components were included for analysis and 99.6% of variance can be explained by the first five principal components. In addition, the PCA revealed that the different feeding groups can be clearly discriminated on the level of the first component indicating a distinct difference of the transcriptomic data. In Fig. 2b the first 20 variables out of the gene data set (loadings) are shown in detail that define the first component. The length of the bar highlights the importance of each variable to contribute to the calculation of the first component. In particular, the gene *OAS1X* revealed to be the most important influencing factor, defining component 1.

The comparison of both feeding groups provided 629 clusters (representing 557 annotated genes) as significantly different (|FC| > 1.5; p < 0.05). Comparing the feeding groups, 156 clusters (= 119 annotated genes) revealed an upregulation and 473 (= 438 annotated genes) a downregulation in the 20% vs. the 10% group (Supplementary Material 1: Table S1). Eight of the top differentially expressed genes were selected for qPCR validation. All of these showed significance (p < 0.05) and high correlations between qPCR and microarray data (Table 1).

**Table 1:** Microarray validation by qPCR

Gene	Fold change (qPCR)	Correlation coefficient r	P-Value
*OAS1X*	-11.6	0.845	0.002
*OAS1Y*	-1.6	0.922	< 0.001
*MX1*	-1.6	0.943	< 0.001
*MX2*	-4.7	0.902	< 0.001
*BOLA*	5.1	0.838	0.002
*ANGPT2*	5.2	0.924	< 0.001
*XCL1*	3.9	0.731	0.016
*GRM8*	4.2	0.678	0.031

**Fig. 3:**
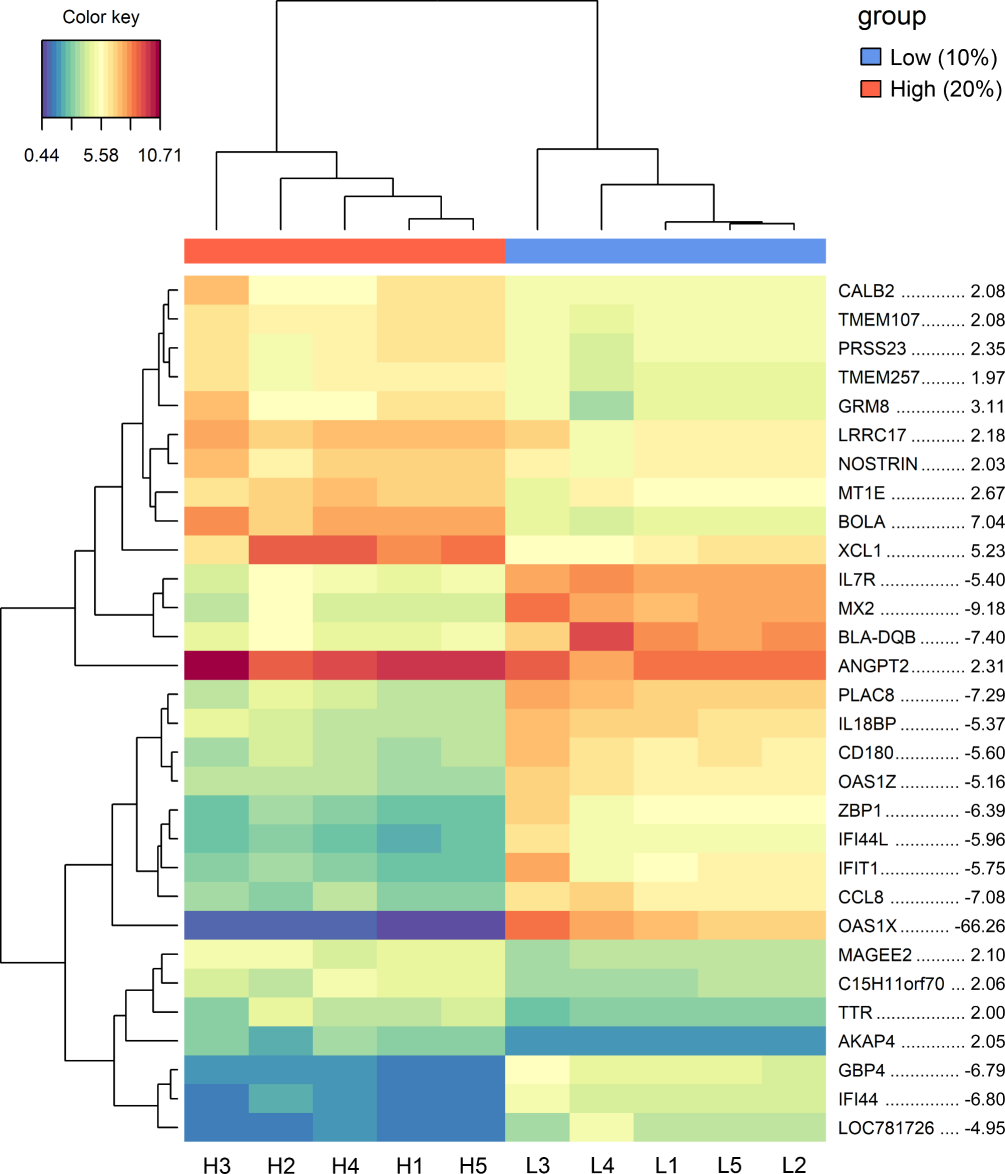
Heatmap and hierarchical clustering of the top 15 up- and downregulated genes. The heatmap visualizes the different signal intensities from low (blue) to high expression (red) for the respective genes in all animal samples. The fold change of displayed genes is shown by comparing the 20% vs. the 10% feeding group

A considerable number of the DEGs were related to immune functions including nearly all known members of the 2,5-oligoadenylate synthetase (OAS) family in cattle. These genes belong to the innate immune system and are induced by interferons [30]. The gene *OAS1X* was by far the strongest downregulated gene in the 20% compared to the 10% feeding group, exhibiting a fold change of -66.26 and very low expression in this group (Fig. 3 and Supplementary Material 1: Table S1). Other downregulated interferon-inducible proteins in the 20% group were the dynamin-like GTPases *MX1* and *MX2* [31]. *BOLA* (FC 7.04) and *XCL1* (FC 5.23) are among the top upregulated genes. *BOLA*, coding for the non-classical MHC class I antigen, is the only gene increased in expression whereas all other classical MHC antigens were downregulated within the 20% group (Supplementary Material 1: Table S2). *XCL1*, also known as lymphotactin α, coding for a small cytokine of the C chemokine family, was 5.23-fold upregulated, whereas the expression of its paralog *XCL2* (lymphotactin β) was not affected.

### Affected pathways identified by Ingenuity Pathway Analysis in the high vs. low feeding group

**Fig. 4:**
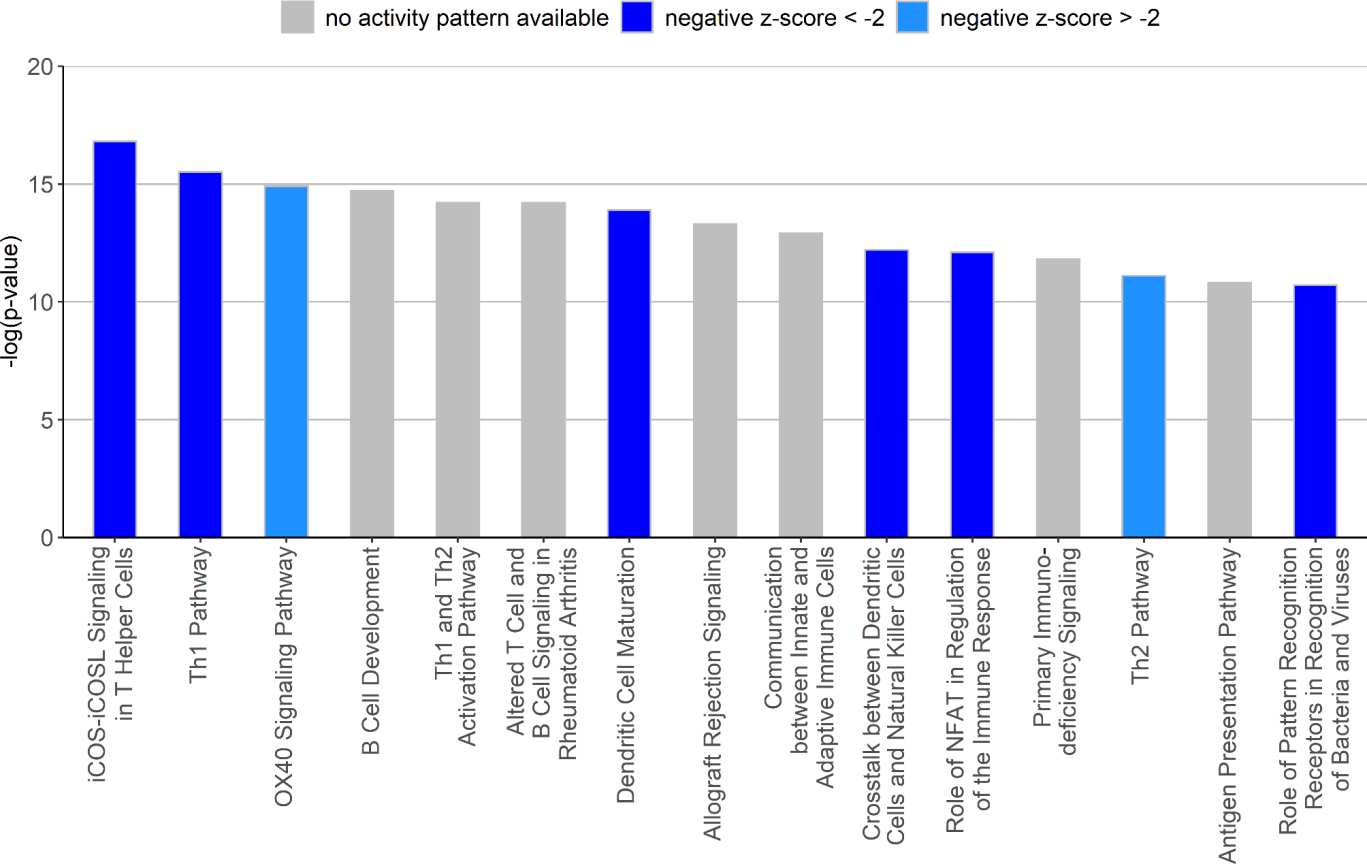
The top 15 affected signaling pathways predicted by IPA. The blue bars represent a predicted suppression of the respective pathway in GCs of the 20% vs. the 10% feeding group indicated by a negative z-score, whereas no prediction could be made for signaling pathways when bars appear grey

**Fig. 5:**
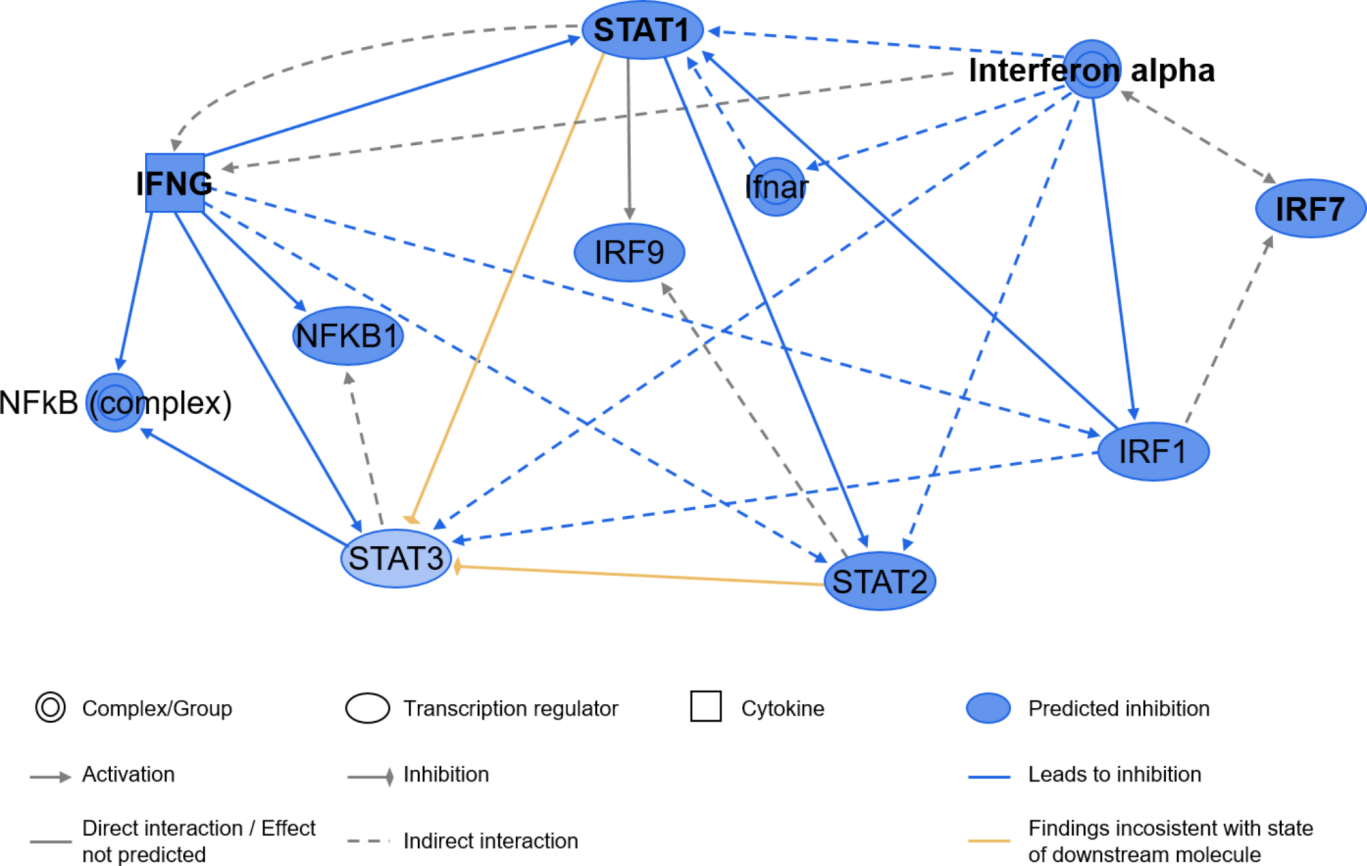
Network of the top upstream regulators Interferon alpha, STAT1, IFNG and IRF7 (bold). The created network was simplified according to the appearance of the different molecules in at least three different upstream regulator networks (modified after analysis by IPA).

Ingenuity pathway analysis revealed several affected immune-related pathways in the GCs of differently fed calves (Fig. 4 and Supplementary Material 1: Table S3). Interestingly, most of these pathways were predicted to be suppressed in the 20% group reflected by a negative z-score. For the signaling pathway “Role of NFAT in Regulation of the Immune Response” the second highest z-score of -4.9 was reported indicating an inhibition in the 20% vs. the 10% feeding group. This pathway included genes coding for major histocompatibility complex proteins as well as surface markers (e.g. CD4). The top 15 predicted upstream regulators are listed in Table 2 including various interferon-related molecules, which inhibit the respective downstream targets (a full table of potential upstream regulators can be found in the Supplementary Material 1: Table S4 including the target genes of respective upstream regulators). The network of Interferon alpha, *STAT1*, *IFNG* and *IRF7* integrates consensus downstream molecules as *STAT3* and *NFKB1* (Fig. 5). The transcription regulators STAT1-3 are mainly involved in the differential regulation of genes of the data set, which were predicted to share mostly inhibitory effects.

**Table 2:** Upstream regulators identified with IPA

Upstream Regulator	Molecule Type	Predicted Activation State	Activation z-score	p-value of overlap
Interferon alpha	group	Inhibited	-6.673	8.45E-47
STAT1	transcription regulator	Inhibited	-5.952	7.82E-44
IFNG	cytokine	Inhibited	-9.072	4.71E-42
IRF7	transcription regulator	Inhibited	-6.607	4.51E-41
IFNA2	cytokine	Inhibited	-6.221	2.71E-37
Ifnar	group	Inhibited	-5.296	7.69E-35
IRF1	transcription regulator	Inhibited	-5.693	1.29E-30
IL4	cytokine		-0.926	4.16E-28
SPI1	transcription regulator	Inhibited	-4.822	6.48E-28
IFNL1	cytokine	Inhibited	-5.177	1.00E-27
TNF	cytokine	Inhibited	-8.639	6.82E-27
STAT3	transcription regulator		-0.444	1.87E-25
IFNB1	cytokine	Inhibited	-5.068	3.33E-25
IL10RA	transmembrane receptor	Activated	6.056	9.24E-25
IFN Beta	group	Inhibited	-4.239	1.02E-24

## Discussion

The BW of the animals differed significantly at the time of slaughter revealing a higher BW of animals fed 20% of milk replacer. BW was also analyzed earlier in life of these animals and differed significantly starting at the fifth week of life [18, 32]. Studies showed that nutrition affects sexual development in heifers and the greatest impact is reported in early postnatal life resulting in an onset of puberty 1–3 months earlier [8]. In our study, however, we did not see a significantly different onset of puberty between both groups which may result from the limited number of animals in both groups and the age at sampling.

The analysis of female sexual organs around puberty is scarce, in particular regarding the development of ovarian structures. In an early study, the negative influence of a low energy diet on the development of ovarian follicles was shown in beef heifers [28]. As expected, calves fed with 20% milk replacer presented an increasing total number of GCs harvested when more follicles < 6 mm could be aspirated. In contrast, the total number of GCs was similar regardless of the number of aspirated follicles in the 10% group, suggesting a control of total GC numbers per ovary irrespective of the follicle count. However, considering transcript concentrations of typical markers of proliferation (e.g. *PCNA*, *MKI67*, or *MCM*) we did not see any regulation among the groups. Our data, however, indicate that the ratio of GCs per oocyte in the 10% group is lower if follicle counts are high. Animals with more than 40 follicles in the 20% group even revealed a 2.4 fold higher ratio of GCs per oocyte. This suggestively should have a considerable impact on oocyte quality. GCs are the main energy and nutrient source for the oocyte and further, GCs are responsible to protect the oocyte against pathogens or free fatty acids [27, 33]. Vice versa, a higher oocyte quality can be assumed in the 20% feeding group due to the higher numbers of GCs per oocyte ratio. This is in accordance with Kelly et al. 2020, who found a larger number of surface follicles and a higher oocyte recovery rate in calves fed a higher plane of nutrition [19]. Since we have no data on oocyte quality yet this must be clarified in a following study.

In a first approach to elucidate molecular bases for this unpredicted limitation of the number of granulosa cells in the 10% group in a genome-wide approach, the transcript levels of the GCs from both feeding groups were compared focusing on five samples with highest follicle counts but very different GC numbers in both groups (see Fig. 1e). According to principal component analysis, a clear separation of the two feeding groups could be observed endorsing the idea that a difference in the plane of nutrition in early life had sustainable effects on GC’s transcriptomes [8]. Among the top regulated differentially expressed genes, *OAS1X*, a member of the OAS family, was the most downregulated gene in the 20% vs. the 10% feeding group. Noteworthy, other members of the OAS system are also found in the list of the DEGs supporting the importance of this gene family. The genes of the OAS family code for the 2’-5’-oligoadenylate synthetases and are involved in the innate immune response to viral infections [30, 34]. In addition, the IFN/OAS/RNase L pathway is linked to cell division regulation and apoptosis [35–37]. Interestingly, several studies connected the expression of *OAS1* with reproductive physiology. *OAS1* contributes to luteolysis by regulating PGF2α [38]. *OAS1* inhibits prostaglandin synthesis and a correlation between decreased PGF2α and increased *OAS1* was described [39, 40] indicating an active cyclic status of calves of the 20% feeding group. A study in mice revealed an association of *OAS1* with reduced fertility in the absence of a protective 2’,5’-OAS enzymatic activity [41]. Consequently, a downregulation of *OAS1* genes in the 20% feeding group might point to a higher oocyte competence associated with a lower 2’,5’-OAS enzymatic activity thus preventing the oocyte and GCs from mRNA degradation and cell death. This hypothesis is additionally supported by the observation of a lower cell number per follicle in the 10% feeding group.

Another top-regulated group of genes are Myxovirus resistance (*MX*) genes, that are dynamin-like GTPases with a broad antiviral activity [42]. These genes are not constitutively expressed but strongly depend on interferon signaling, thus being a reliable and direct marker for IFN action [43, 44]. In the bovine, at least two paralogs have been identified, *MX1* and *MX2*, that are expressed in the endometrium during pregnancy [45]. We observed higher levels of both genes in the 10% group thus indicating an active interferon action in this group.

*BOLA*, coding for the non-classical MHC class I antigen, is the only gene revealing an increase of expression in the 20% feeding group whereas all other classical MHC antigens show a downregulation. The expression of classical MHC class I in granulosa cells results in follicular atresia and subsequently to premature ovarian failure [46], whereas the presence of MHC class II antigens in GCs revealed to be necessary for the luteal transition of these cells [47]. Hence, the higher expression of classical MHC genes in the 10% feeding group suggests an abnormal ovarian function in this group.

Ingenuity Pathway Analysis revealed several pathways affected which can be grouped together as ‘immune-relevant’ pathways. If predicted, mostly an inhibition of the respective pathway was indicated in the 20% group.

The pathway “Role of NFAT in Regulation of the Immune Response” was predicted to be most strongly suppressed in the 20% group emphasizing the importance of NFAT in the regulation of immune responses. An expression in ovary of members of the NFAT family was identified before [48–50]. Although the expression of different NFAT factors was not altered, several receptors and kinases involved in NFAT signaling (*PCLB2*, *ITK*, *PIK3CG*) revealed a substantial downregulation in the 20% feeding group. NFAT is a family of transcription factors involved in many developmental processes, e.g. skeletal muscle development, development and function of the cardiovascular system, bone homeostasis and inflammatory responses [51, 52]. Moreover, calcineurin/NFAT signaling pathway induce COX-2 gene expression in endometrial stromal cells of rat [53]. The expression of COX-2 is additionally crucial for the fine-tuned process of ovulation within the follicle. Hence, the calcineurin/NFAT pathway might be another important element within the accurate progression of folliculogenesis when a high impact of this signaling cascade is revealed as a result of the different feeding regimes.

From the analyses of the signaling pathways affected, it is not surprising that interferons are predominant among the top upstream regulators. In this list, interferon alpha and gamma are only two examples, again supporting the hypothesis of deregulated immune response within the 10% feeding group. The involvement of interferon in GC physiology was described earlier when the proliferation capacity of porcine GCs decreased with higher concentrations of IFNα [54]. IPA predicted the inhibition of interferon alpha in the 20% feeding group compared to the 10% group. According to the results of Yasuda et al. [54], increased interferon alpha concentrations inhibit GC proliferation in a dose dependent manner. Considering this for the 10% feeding group, it might be a possible explanation for the constantly low numbers of GCs, even at high follicle counts. Additionally, interferon γ was shown to inhibit progesterone and estradiol production in human luteinized GCs or cultured rat GCs [55, 56]. Besides different interferons, several transcription factors are highlighted as regulators responsible for the transcriptional changes in the 20% vs. the 10% feeding group. In the generated regulator network, besides interferon, the family of STAT transcription factors is mainly involved. The expression of *STAT1* is downregulated in the 20% group and additionally acts as inhibiting regulator of the differentially expressed genes. The STAT transcription factors are activated by interferons [57, 58] and are fundamental parts of the JAK/STAT signaling pathway that is known to be involved in cell proliferation and in the regulation of all stages of follicle development [59]. Interestingly, overexpression of STAT1 has inhibitory effects on the expression of proliferation markers and promoting markers of apoptosis in porcine GCs [60]. Although we did not see a direct effect on markers of proliferation or apoptosis, a similar regulation might be present within the 10% feeding group. Hence, the different feeding regimes in early life might influence general signaling pathways in the ovary as well as GC function in particular.

## Conclusions

In conclusion, different feeding regimes in early life significantly affect the GC per oocyte ratio, and GCs’ transcriptomes in peripubertal heifers. It is reasonable to assume that these changes might further affect oocyte competence, which in turn has implications for overall reproductive life (e.g. fertility) in cattle. As a number of interferon dependencies and immune-relevant pathways were detected, we cannot rule out the possibility that a different immune profile or immune sensitivity exists in GCs of both groups. Interestingly, this is set in an early stage of life and can be seen even months later, highlighting a nutritional programming occurring in the sensitive phase of early life.

## Methods

### Animal model, tissue sampling and isolation of granulosa cells

Data of this study are from 22 female German-Holstein calves. The experiment was conducted at the Research Institute for Farm Animal Biology in Dummerstorf, Mecklenburg-Vorpommern, Germany. The calves were purchased directly after birth from local commercial farms and randomly assigned into two experimental groups without receiving any colostrum at the farm of origin. In the 10% group, the calves (n = 11) were fed with a maximum of 10% milk replacer/kg bodyweight (BERGIN Milch LC 50, Bergophor Futtermittelfabrik Dr. Berger GmbH & Co. KG, Kulmbach, Germany; 140 g of powder/kg; DM (%) 95.0 ± 1.3; 17.0 MJ/kg of DM). In contrast, the calves of the 20% group (n = 11) received a maximum of 20% milk replacer/kg bodyweight (BERGIN Milch LC 50) per day from an automatic calf feeder (Kälbermama Lifestart, Urban GmbH & Co. KG, Hude/Wüsting, Germany). The milk allowance was adjusted weekly after weighing. The 10% group received the whole milk replacer allowance for ten weeks followed by a two-week transition period from milk feeding to sole solid feeding. During the transition period, the daily milk allowance was gradually reduced. For the 20% group the transition period started already in week eight but lasted for four weeks. For both experimental groups milk feeding stopped after week 12. During the first 12 weeks of life, the calves of both groups were able to consume hay and calf starter ad libitum. Hay ad libitum feeding was continued until the end of week 14. The calf starter was limited to 2 kg/day in week 13 to 14 and thereafter gradually reduced until the end of week 16. From week 11 onwards, all calves additionally received a total-mixed ration ad libitum. After week 16, the total-mixed ration was the sole feed offered for ad libitum intake in both groups until sampling (Supplementary Material 2). For further detailed information on the nutritional plan, feed analysis and colostrum feeding see Tümmler et al. [18, 32]. Additionally seven animals per group were equipped with a rumen cannula in week three of age.

All 22 animals were sacrificed between the age of 33 and 43 weeks (mean: 34.9 weeks) in the institute-owned slaughterhouse. The ovaries were recovered directly afterwards and transported to the laboratory in 1× PBS solution (supplemented with 100 IU penicillin, 0.1 mg/ml streptomycin and 0.5 µg/ml amphotericin; Bio&Sell, Feucht, Germany) for GC preparation within minutes.

GC preparation started immediately after arrival at the laboratory. All follicles smaller than 6 mm in diameter were aspirated with a syringe and 18 G needle by a single experienced technician without knowing the assignment to the experimental group. Subsequently, living cells were counted by the trypan blue exclusion method in a haemocytometer, by the same technician. GCs were gently centrifuged (500 × *g*, 3 min) to prevent cell death and RNA degradation. The cells were cryo-preserved in freezing media (fetal calf serum containing 10% DMSO; Roth, Karlsruhe, Germany) and stored in the Corning® CoolCell™ Container at -80 °C to guarantee a controlled temperature decrease of 1 °C per minute ensuring that the cells remain alive for further analysis [61].

### RNA preparation, mRNA microarray profiling, bioinformatics evaluation and statistics

For RNA isolation, cells were thawed and immediately separated from the preservation medium by centrifugation (500 × *g*, 3 min) and lysed with the respective buffer of the isolation kit. Isolation of total RNA was conducted with the RNeasy Kit (Qiagen, Hilden, Germany) according to the manufacturer’s instructions. Subsequently, RNA concentration was measured with a NanoDrop 1000 Spectrophotometer (Thermo Scientific, Bonn, Germany). The quality of RNA was checked in a Bioanalyzer Instrument (Agilent Technologies, St. Clara, CA, USA) showing RIN factors ranging from 7.7 to 9.1. For the transcriptome analysis, GCs of five calves with highest follicle but different GC counts in each feeding group were selected (10% group: L1 to L5; 20% group: H1 to H5). The selection criterion was 40 or more follicles. The microarray analysis was performed with the Bovine Gene 1.0 ST Array (Affymetrix, St. Clara, CA, USA) representing more than 26,000 transcript clusters. The amplification procedure, labelling and hybridization was accomplished with the “GeneChip Expression 3’ Amplification One-Cycle Target Labeling and Control Reagents” (Affymetrix) following the manufacturer’s protocol. Overnight hybridization was done in the GeneChipR Hybridization Oven (Affymetrix) and visualized with the Affymetrix GeneChip Scanner 3000. The obtained raw data files were processed in the Transcriptome Analysis Console 4.0.2 (TAC4.0.2, Affymetrix). First, influencing effects (= batch effects) were considered for the analysis including the presence of a rumen fistula, the age at slaughter and the follicle count. The impact of the batch effects on differentially expressed genes was calculated with the TAC4.0.2 software and revealed to be 3%, 2% and 2%, respectively. Second, normalization, background reduction and a gene-level summary was done using the implemented RMA method (Robust Multichip Average) followed by principal component analysis (PCA) using the mixOmics package in R [62]. Subsequently, analysis for differentially expressed genes (DEGs) of the two different feeding groups (20% vs 10%) was carried out with the following criteria of significance of fold change (FC) > |1.5| and p < 0.05. False discovery rates (FDR) were computed with the Benjamini-Hochberg method but were not used as cut-off criterion in the study. Statistic evaluation of additional data was performed with SigmaPlot 11.0 Statistical Analysis System (Jandel Scientific, San Rafael, CA, USA). The threshold for significance was set at p < 0.05. The subsequent functional annotation was performed with Ingenuity Pathway Analysis (IPA, Qiagen). Thus, an extended list of 1458 genes with FC > |1.2| and p < 0.05 was imported into IPA and 1056 genes were mapped. For the actual pathway analysis the fold change threshold was set to |1.5|, still showing genes with |1.2| < FC < |1.5| as abundant in the dataset.

### cDNA synthesis and qPCR validation

The microarray data were validated with quantitative Real-Time PCR (qPCR). Therefore, cDNA was synthesized with the SensiFAST cDNA Synthesis Kit (Bioline, Luckenwalde, Germany) from 150 ng RNA (Supplementary Material 1: Table S5). For this approach the SensiFAST SYBR No-ROX Kit (Bioline) was used in combination with gene-specific primers as listed in Table 3. Amplification of genes of interest was conducted in a LightCycler 96 instrument (Roche, Mannheim, Germany) in duplicate from 0.2 to 0.4 µl cDNA in a total volume of 12 µl. The following cycle conditions were applied: pre-incubation at 95 °C for 5 min, 40 cycles of amplification of denaturation (95 °C, 20s), annealing (60 °C, 15 s) and extension (72 °C, 15 s) and a single-point fluorescent acquisition for 10 s. Melting point analysis was done after completion of each run to check amplification of intended target products. Additionally, PCR products were evaluated by agarose gel electrophoresis (3%, stained with Roti-GelStain, Roth, Karlsruhe, Germany). For quantification, external standards for each gene of interest were generated by cloning and checked by sequencing. Accordingly, five different dilutions of the particular standard (5 × 10^− 12^ − 5 × 10^− 16^ g DNA/reaction) were freshly prepared and co-amplified. *B2M* (Beta-2-microglobulin), *GAPDH* (Glyceraldehyde-3-phosphate dehydrogenase), *RPLP0* (Ribosomal protein lateral stalk subunit P0) and *TBP* (TATA box binding protein) were utilized as potential reference genes; the geNORM algorithm implemented in the NormqPCR package for R [63] identified *RPLP0* and *TBP* as suitable gene for normalization, the geometric mean of both was used.

**Table 3:** Gene specific primers for qPCR

Gene	direction	Sequence	Size (bp)	Accession No.
*RPLP0*	for	TGGTTACCCAACCGTCGCATCTGTA	142	NM_001012682
	rev	CACAAAGGCAGATGGATCAGCCAAG		
*TBP*	for	GCCTTGTGCTTACCCACCAACAGTTC	200	NM_001075742
	rev	TGTCTTCCTGAAACCCTTCAGAATAGGG		
*B2M*	for	ACGCTGAGTTCACTCCCAACAGCAA	114	NM_173893
	rev	TCGATGGTGCTGCTTACAGGTCTCG		
*GAPDH*	for	AGCGAGATCCTGCCAACATCAAG	221	NM_001034034
	rev	GCAGGAGGCATTGCTGACAATCT		
*BOLA*	for	CAGCGACGCCCCGAATCCGAGAA	201	XM_005223701
	rev	CCCACTTCGCAGCCAAACATCACCT		
*OAS1X*	for	ACGTGAAGCTCATCCAAGAGTGCGAGA	207	NM_178108
	rev	AGACGGTCAGCAGCTCCAGGGCAT		
*OAS1Y*	for	TTCTGGACCCGGCGGACCCT	153	NM_001040606
	rev	CTTGGGGCGACACATCCCAGGAGC		
*MX1*	for	GGGACGGGCGGTGTTGGAGCA	250	NM_173940
	rev	ACGCCCAGGGACCGCAGGGA		
*MX2*	for	TGAAGTGCCGGGGCCAGCAGGATA	237	NM_173941
	rev	GCTCCTCTGTCGCCCTCTGGTGCT		
*ANGPT2*	for	TGGAAACGGGTGGAGGTGGGTGGA	289	NM_001098855
	rev	CCGGCTGTCCCCGTGAGGCCTTTA		
*XCL1*	for	TGCCTGGTGATCTGCAGTCTCGCT	270	NM_175716
	rev	GGCTCCTGTAGGCTTGGCCTGGCT		
*GRM8*	for	TGGTGTTATCGGTGCTGCCGCGAG	200	NM_001206117
	rev	ACCCCAGGGCCGTCACAATGTCCA		

for, forward primer; rev, reverse primer

### Electronic supplementary material

Below is the link to the electronic supplementary material.

Supplementary Material 1

Supplementary Material 2

## Data Availability

The generated microarray dataset is available in the GEO database with the following Accession: GSE216927. The remaining datasets are available from the corresponding author on request.

## Abbreviations

*ANGPT2*: Angiopoietin-2
*B2M*: Beta-2-microglobulin
*BOLA*: Non-classical MHC class I (bovine)
BW: Body weight
CL: Corpus luteum
COX-2: Cyclooxygenase 2
DEG: Differentially expressed genes
DMSO: Dimethyl sulfoxide
FC: Fold change
*GAPDH*: Glyceraldehyde-3-phosphate dehydrogenase
GC: Granulosa cell
*GRM8*: Metabotropic glutamate receptor 8
IFN: Interferon
IPA: Ingenuity Pathway Analysis
*IRF7*: Interferon regulatory factor 7
MHC: Major histocompatibility complex
MR: Milk replacer
*MX1*/*MX2*: Myxovirus resistance genes
NFAT: Nuclear factor of activated T-cells
*NFKB1*: Nuclear factor NF-kappa-B
*OAS*: 2’-5’-oligoadenylate synthetases family
PBS: Phosphate buffered solution
PCA: Principal component analysis
*RPLP0*: Ribosomal protein lateral stalk subunit P0
*STAT*: Signal transducer and activator of transcription
*TBP*: TATA box binding protein
*XCL1*/*XCL2*: Chemokine (C motif) ligand
