# Supplementary figures and images for: Early milk-feeding regimes in calves exert long-term effects on the development of ovarian granulosa cells
Source: BMC Genomics. 2023 Aug 25;24:485. doi: 10.1186/s12864-023-09589-7 (PMC10464335; doi:10.1186/s12864-023-09589-7)

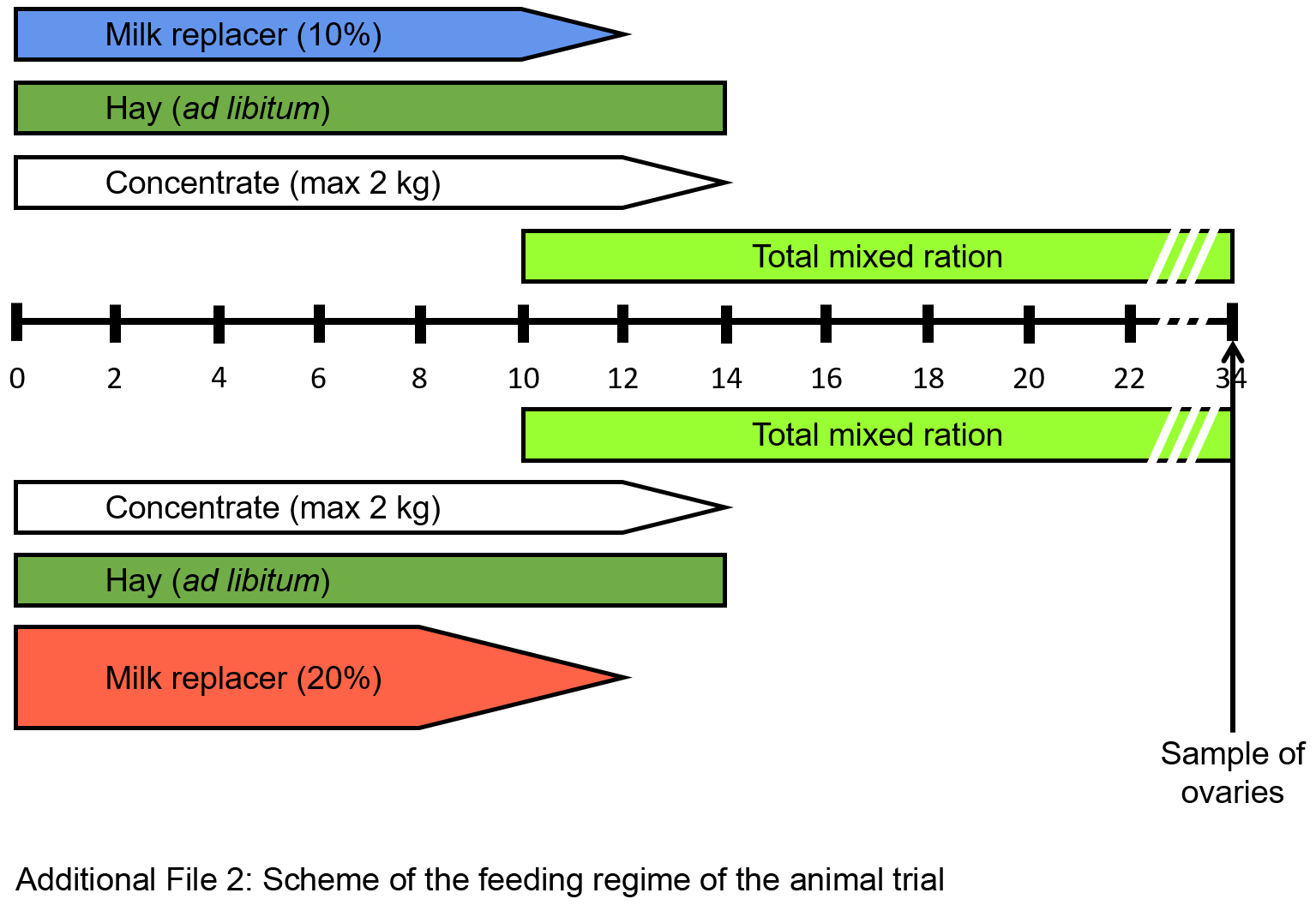

Supplement: Supplementary file 2 — Supplementary Material 2 [file 12864_2023_9589_MOESM2_ESM.tif]
